# The Acute Effects of Heavy Sled Towing on Acceleration Performance and Sprint Mechanical and Kinematic Characteristics

**DOI:** 10.3390/sports10050077

**Published:** 2022-05-16

**Authors:** Maria Zisi, Ioannis Stavridis, Georgia-Olanemi Agilara, Theodosia Economou, Giorgos Paradisis

**Affiliations:** Sports Performance Laboratory, School of Physical Education & Sport Science, National & Kapodistrian University of Athens, Ethnikis Antistaseos 41, 172 37 Dafni, Greece; zismaria3@gmail.com (M.Z.); ioastavridis@phed.uoa.gr (I.S.); georgia_bs@windowslive.com (G.-O.A.); thiconom@gmail.com (T.E.)

**Keywords:** post-activation potentiation, resisted sprinting, acceleration, performance, sprint kinematics, sprint mechanics

## Abstract

The aim of this study was to investigate the effects of heavy sled towing using a load corresponding to a 50% reduction of the individual theoretical maximal velocity (ranged 57–73% body mass) on subsequent 30 m sprint performance, velocity, mechanical variables (theoretical maximal horizontal force, theoretical maximal horizontal velocity, maximal mechanical power output, slope of the linear force–velocity relationship, maximal ratio of horizontal to total force and decrease in the ratio of horizontal to total force) and kinematics (step length and rate, contact and flight time). Twelve (*n* = 5 males and *n* = 7 females) junior running sprinters performed an exercise under two intervention conditions in random order. The experimental condition (EXP) consisted of two repetitions of 20 m resisted sprints, while in the control condition (CON), an active recovery was performed. Before (baseline) and after (post) the interventions, the 30 m sprint tests were analyzed. Participants showed faster 30 m sprint times following sled towing (*p* = 0.005). Running velocity was significantly higher in EXP at 5–10 m (*p* = 0.032), 10–15 m (*p* = 0.006), 15–20 m (*p* = 0.004), 20–25 m (*p* = 0.015) and 25–30 m (*p* = 0.014). No significant changes in sprint mechanical variables and kinematics were observed. Heavy sled towing appeared to be an effective post-activation potentiation stimulus to acutely enhance sprint acceleration performance with no effect on the athlete’s running technique.

## 1. Introduction

Sprint acceleration is an important component in running performance. The sprint acceleration phase is characterized by the capacity to produce high levels of force and power in order to reach the maximum running velocity [1]. Acceleration performance depends on sprint kinetics, such as the magnitude and the orientation of the ground reaction force vector [2]. Mechanical effectiveness, i.e., the effective application of lower limb force in a horizontal direction as velocity increases, is significantly related to sprint performance [3,4,5]. High-level sprinters are able to produce greater horizontal force and impulse throughout the acceleration phase compared to lower-level sprinters [6].

The ability to apply horizontally oriented force can be determined by the force–velocity (F-v) and power–velocity (P-v) relationships [3]. The horizontal F-v profile can be easily estimated using inverse dynamics applied to the athlete’s body center of mass (CM) during sprint acceleration, including theoretical maximal horizontal force (*F*_0_) and velocity (*v*_0_), maximal mechanical power output (*P_max_*), the ratio of force (*RF*) which expresses mechanical effectiveness and *D_RF_* which describes the rate of decrease in *RF* as the velocity increases over the entire acceleration phase [3,7].

Various training modalities have been developed to produce acute and chronic improvements in athletes’ ability to accelerate rapidly [8,9,10,11]. Post-activation potentiation (PAP) is a worthwhile method to acutely enhance performance in explosive activities, such as sprinting [9], jumping [12] and throwing [13]. Many studies showed improvements in sprint performance using resistance exercises [11], resistance running [9] and plyometrics [14] as conditioning stimuli in a PAP protocol. The response to a PAP protocol depends on methodological factors, including the type and the intensity of muscle contraction, the length of the rest period, the training background of the participants and their physiological characteristics [15,16,17]. Finally, previous researchers highlighted the importance of mechanical similarity between conditioning stimuli and subsequent activity [18].

Resisted sprints using weighted sleds is a sprint-specific training method to improve performance during the acceleration phase [19,20]. It is reported that during sled towing, an acute improvement in force application occurs, and mechanical effectiveness is enhanced as the load increases [21]. Additionally, researchers have shown that heavier loads increase contact time and propulsive duration, resulting in greater horizontal force application [22]. Recently, Cross et al. [23] found that the optimal load to maximize power production during sled resisted sprints was approximately a 50% decrement of *v*_0_ (ranging from 69 to 96% of body mass (ΒΜ) in recreational athletes and sprinters). This finding indicates that defining a load as a decrement in sprint velocity (v_dec_) might be a better approach to determining individual load instead of the percentage of BM.

Weighted sled towing with a wide range of loads is used to acutely enhance acceleration performance [9,24,25,26,27,28,29]. Recent studies have applied heavier loads to induce PAP. Jarvis et al. [27] found that resisted sprints of 50% BM improve subsequent 15 m sprint performance after 8 min of recovery. Winwood et al. [9] examined the acute effect of sled towing with loads of 75 and 150% BM on 15 m sprint performance and found that performance enhancement occurred with a load of 75% BM. Similarly, another study indicated faster 20 m sprint times after sled pushing with a load of 75% BM, but not after 125% BM in male rugby players [30]. An explanation for the inefficiency of loads > 125% BM is the prevalence of fatigue after such high stimuli [9].

Sprint kinematics, including the step length and frequency and the flight and contact time, determine the development of the velocity during sprinting [10]. Studies indicated that during sled towing, velocity is impaired due to the reduction of step length, step frequency and flight time and the increase of contact time [19,31]. Nevertheless, sled towing training for 6 weeks enhanced sprint velocity, mainly by increasing the step length [10]. Studies that investigated the acute effects of resisted sprints on kinematics are limited and have failed to demonstrate changes in the kinematic characteristics of the subsequent sprint [24,26,27,28,29]. Furthermore, to our knowledge, no study has examined the acute effects of heavy sled towing on sprint kinematics throughout the acceleration phase. 

Horizontal application of force and alterations in sprint kinematics affect performance during the sprint acceleration phase [4,10]. Heavy sled towing is a sprint-specific activity which increases horizontal force output [21,22] and maximizes power output when loads of 50% v_dec_ are applied [23]. Furthermore, sled towing may cause long-term improvements in sprint kinematics, especially in step length, but there is no evidence of acute effects [10]. Therefore, the objective of this study was to examine whether resisted sprints with individual load causing a 50% v_dec_ of *v*_0_ could improve 30 m sprint performance by causing changes in sprint mechanical characteristics. Additionally, the acute effects of sled towing with the individual optimal load on sprint kinematics were investigated. It was hypothesized that resisted sprints with 50% v_dec_ load would improve 30 m sprint performance and underlying mechanical variables.

## 2. Materials and Methods

### 2.1. Experimental Design

A randomized, crossover and counterbalanced experimental design was applied to investigate whether resisted sprints could affect 30 m sprinting performance, mechanical variables (*F*_0_, *v*_0_, *P_max_*, *S_Fv_*, *RF_max_* and *D_RF_*), sprinting kinematics (step length and frequency, contact and flight time) and running velocity per 5 m. The sled load used corresponded to a 50% v_dec_ of the individual’s *v*_0_, which is considered to be the optimal load causing the maximum power production [23]. The participants completed 4 sessions on non-consecutive days, separated by at least 48 h. The first session included the determination of the individualized sled-towing load which would be used as the conditioning stimulus. During the second session, participants were familiarized with their individual load on sled towing, experimental protocols and testing procedures. In sessions 3 and 4, participants performed under one of two intervention conditions randomly, (1) control condition (CON), which consisted of a standardized warm-up or (2) experimental condition (EXP), in which a standardized warm-up was followed by sled towing sprints. All sessions were conducted in a synthetic track at the same time of day in order to avoid the possible effects of testing time. The participants wore their tracking footwear during each session and were asked to refrain from intensive exercise for 24 h before the tests. 

### 2.2. Participants

Twelve junior sprinters, five males and seven females (age: 17.2 ± 1.6 years; body mass: 65.4 ± 8.8 kg; stature: 1.75 ± 0.07 m), volunteered to be included in this study. Exclusion criteria included musculoskeletal diseases or injuries that could affect the experimental processes. To verify the sample size of this study, a statistical power calculation was performed. The sample size was adequate for the variables with significant interactions or main effects (a ≤ 0.05 for type I error), whereas it was not adequate for the variables with no significant interactions or main effects (b ≤ 0.2 for type II error). This is considered a research limitation. Participants had at least three years of training experience and were familiar with loaded sled towing. All participants were informed about the experimental procedures and the potential risks and benefits of their involvement in the study and gave their written consent. Parental consent was required for participants under 18 years of age. The study protocol was conducted in accordance with the Declaration of Helsinki and was approved by the local ethical committee. 

### 2.3. Procedures

At the beginning of each session, participants performed a standardized warm-up consisting of jogging, dynamic stretching of the muscles of the lower limbs, sprint-specific drills and three progressive 30 m sprints. Recovery between the three progressive sprints was a 2 min walk. In the first session, 5 min after warm-up, participants performed one unresisted and three resisted 30 m maximal sprints towing a sled with a load corresponding to 25, 50 and 75% of BM in order to determine the load-velocity relationship [23,32]. The peak running velocity was obtained for unresisted and resisted sprint trials. A 5 min recovery was performed between trials. The load–velocity data were fit with a least-square linear regression to compute an individualized load–velocity profile for each participant. The computation of optimal load was performed using a validated Excel spreadsheet, based on the reduction of velocity through a linear load–velocity relationship as the load increased [23,32]. Sprint trials were recorded by a high-speed panning camera placed at 15 m. Before intervention sessions, participants were familiarized with the experimental protocols, individualized sled load and testing procedures. In testing sessions, 5 min after performing a standardized warm-up [33], participants performed a baseline 30 m sprint from a three-point crouching position followed by the execution of an intervention condition. In EXP, 5 min after the baseline trial, 2 repetitions of 20 m resisted sprints with individualized load were completed with 2 min of recovery between the sprints [19,27,29]. A sled sprint distance of 20 m for the EXP condition was used to standardize the time under a tension of ~5 s in order to maintain the maximum horizontal power [32]. CON consisted of active recovery (walking) of the same duration as the conditioning exercise. After completing the protocols, participants completed a 30 m sprint at 8 min of recovery. During sprint trials, all participants were verbally encouraged to maximum effort in the entire 30 m distance. During the testing sessions, sprint trials were captured using 3 high-speed panning cameras (Casio EX-F1, Tokyo, Japan) sampling at 300 Hz. Cameras were placed on tripods in the sagittal plane, 10 m away from the middle of the running lane. The placement of the cameras was at 5, 15 and 25 m of the 30 m trial, recording the 0–10, 10–20 and 20–30 m of the sprint, respectively [31]. Split times of 5, 10, 15, 20, 25 and 30 m were determined using marking poles which were placed along the distance of 30 m sprint, ensuring the correction of video parallax error [34]. Additionally, in order to measure step length, 0.05 m × 0.05 m custom markers were placed on both sides of the running lanes across the entire runway [35].

### 2.4. Data Analysis

The split times per 5 m were determined by analyzing the video data using Kinovea software (v.0.8.15). The frames in which the hip of the athlete crossed the marking poles defined the six different split times, and running velocity was calculated for every 5 m interval. The starting point of the sprint was defined as the first propulsive movement of the rear leg from the three-point crouching position. Kinematic analysis was performed for each step using Quintic Biomechanics software (v31) (Quintic Consultancy Ltd., Birmingham, UK). The frame of touchdown and toe-off of each step was determined. The time the foot touched the ground was calculated as the contact time. The time between the toe-off of one foot to the touchdown of the other foot was calculated as the flight time. Step length was defined as the distance between the toes of two consecutive steps. For each step, the distance between the toe of the athlete’s foot and the custom markers was computed by extending a line between the point of toe during touchdown and the distance between the near pairs of markers [36]. The step rate was calculated as the running velocity/step length. All variables analyzed were presented as the average values per 5 m splits. For each unresisted trial, baseline and post-intervention 30 m sprints were analyzed in order to determine the components of the sprint mechanical profile (*F*_0_, *v*_0_, *P_max_*, *S_Fv_*, *RF_max_* and *D_RF_*) according to Samozino’s method [3]. The intraclass correlation coefficient between baseline trials, based on 30 m sprint performance, was very high (0.994, 95% confidence interval from 0.978 to 0.998).

### 2.5. Statistical Analysis

All data are presented as mean ± standard deviation (*SD*). The Shapiro–Wilk test was used to confirm the normality of the data. A two-way repeated measures ANOVA (2 conditions: control, experimental × 2 times: pre, post) was conducted to test the significance of differences in sprint time, running velocity and kinematic variables (step length and rate, contact and flight time) at each interval distance and mechanical components (*F*_0_, *v*_0_, *P_max_*, *S_Fv_*, *RF_max_*, *D_RF_*) between baseline and post-intervention trials. Interactions and main effects were examined, and Bonferroni post hoc analysis was applied when statistical significance was observed. Cohen’s d effect size (ES) was used to determine the magnitude of the within-subjects effect. Effect size was classified as: trivial (<0.20), small (0.20 to 0.49), medium (0.50 to 0.79) or large (≥0.80) [37]. Statistical significance was determined by an alpha level of *p* ≤ 0.05. Statistical Software SPSS (IBM SPSS version 25.0, Chicago, IL, USA) was used for all analyses.

## 3. Results

The results of the ANOVA indicated a significant interaction effect (condition × time) for the 30 m sprint time (F = 5.85, *p* = 0.034, η^2^ = 0.35, observed power (OP) = 0.60). Post hoc analysis revealed that the EXP was significantly faster in the post-intervention 30 m sprint compared to the baseline trial (pre = 4.68 ± 0.38 s, post = 4.64 ± 0.37 s, mean difference (MD) = −0.047 ± 0.013 s, *p* = 0.005, ES = 0.48). No significant difference was observed in CON (pre = 4.65 ± 0.37 s, post = 4.66 ± 0.35 s).

**Figure 1:**
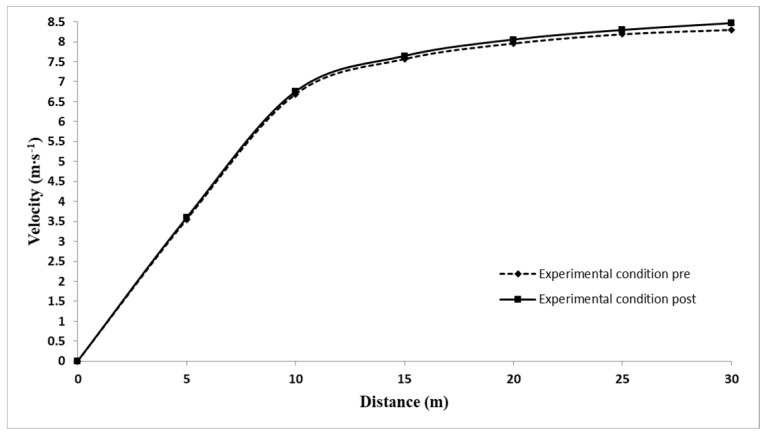
Velocity of baseline and post-intervention 30 m sprints for the experimental condition.

An interaction effect (condition x time) was noticed in running velocity at 5–10 (F = 5.46, *p* = 0.039, η^2^ = 0.33, OP = 0.57), 10–15 (F = 5.07, *p* = 0.046, η^2^ = 0.32, OP = 0.54), 15–20 (F = 5.62, *p* = 0.037, η^2^ = 0.34, OP = 0.58), 20–25 (F = 5.86, *p* = 0.034, η^2^ = 0.40, OP = 0.68) and 25–30 m (F = 7.15, *p* = 0.022, η^2^ = 0.40, OP = 0.75) distance intervals. Post hoc interaction effect analysis indicated that the EXP achieved significantly higher running velocity following resisted sprints at 5–10 (MD = 0.06 ± 0.03 m∙s^−1^, *p* = 0.032, ES = 0.71), 10–15 (MD = 0.08 ± 0.02 m∙s^−1^, *p* = 0.006, ES = 0.98), 15–20 (MD = 0.08 ± 0.02 m∙s^−1^, *p* = 0.004, ES = 1.06), 20–25 (MD = 0.10 ± 0.03 m∙s^−1^, *p* = 0.015, ES = 0.83) and 25–30 m (MD = 0.09 ± 0.03 m∙s^−1^, *p* = 0.014, ES = 0.84) (Table 1, Figure 1). CON did not show any significant difference in running velocity (Table 2). No significant interaction and main effect were found in running velocity at 0–5 m distance interval for both EXP and CON. 

No significant interaction, main time and condition effect were observed in the sprint mechanical variables *F*_0_, *v*_0_, *P_max_*, *S_Fv_*, *RF_max_* and *D_RF_* for both conditions (Table 3). However, when assessing the within-subjects changes of baseline and post-intervention trials, there were some trends of increased *v*_0_ (ES = 0.43) and *P_max_* (ES = 0.47) in EXP. Sprint kinematic variables for EXP and CON are presented in Table 1 and Table 2. There were no significant (*p* > 0.05) interaction effects or main time and condition effects in step length, step rate, contact and flight time.

## 4. Discussion

The purpose of this study was to examine the potentiating effects of heavy sled towing on subsequent 30 m sprint performance and on sprint mechanical and kinematic characteristics. The load used, ranging from 57 to 73% BM, corresponded to a 50% v_dec_ of individual *v*_0_, which was proposed as an optimal load to maximize power production during sled towing [23]. In accordance with the hypothesis, the main finding of this study was that 30 m sprint performance was acutely improved by 1% following two repetitions of sled towing. All participants except one male produced faster 30 m sprint times. Additionally, athletes’ sprint velocity increased following resisted sprints by 0.92, 0.98, 1.05, 1.17 and 1.07% at distances 5–10 m, 10–15 m, 15–20 m, 20–25 m and 25–30 m, respectively.

The results of this study support the findings of previous literature. Winwood et al. [9] found improvements in 15 m sprint performance in resistance-trained rugby players following sled towing with a load of 75% BM (eliciting a v_dec_ by 23–37%). However, due to the individual variability in v_dec_ caused by the load, the researchers suggested the v_dec_ as a better method of determining the sled load. Williams et al. [25] also indicated that heavy sled towing potentiates 15 m sprint performance in young male and female football players. The sled load used in this study was equivalent to 40–50% v_dec_ (ranged from 66 to 70% of BM) and elicited faster sprint times by an average of 0.1 s. Monaghan and Cochrane [24] investigated whether forward and backward sled towing with loads of 35 and 55% v_dec_ would affect 5 m sprint performance in well-trained rugby male players. In contrast to previous findings, researchers found no significant change in sprint velocity. Similarly, Whelan et al. [29] showed no significant effect on 5 m and 10 m sprint velocity in physically active males using a sled load of 25–30% BM. The results of these studies are consistent with the present study since no significant improvement in velocity was found at 5 m sprint. It can be assumed that such a short distance may not be sufficient to observe a potentiating effect.

Heavy sled towing creates an overload effect that appears to be effective in the acute enhancement of performance during the acceleration phase if adequate recovery is provided. Sheitz and Haff [38] indicated that a recovery time > 5 min is required after the execution of moderate and high resistance exercises. The 8 min recovery between conditioning activity and subsequent performance used in this study was sufficient to minimize fatigue and elicit the PAP effect. This finding is consistent with previous research, which demonstrated faster 15 m sprint times following resisted sprints with a load of 50% BM and 8 min of recovery [27].

The individual sled load used in this study was sufficient to activate the responsible mechanisms for the PAP phenomenon. A possible mechanism for the enhancement of sprint performance is the increase of neural activation and recruitment of higher-order motor units [16,39]. Due to the absence of electromyography in the current study, it can only be assumed that heavy sled towing caused the activation of type II muscle fibers specific to sprinting, which resulted in higher force and power production. Another potential explanation for improved performance is the phosphorylation of myosin regulatory light chains that increases the sensitivity of the actin–myosin complex to calcium (Ca^2+^), leading to an increased rate of binding of myosin cross-bridges to the actin [16]. The present results showed that there were no significant changes in the mechanical properties *F*_0_, *v*_0_, *P_max_*, *S_Fv_*, *RF_max_* and *D_rf_* after performing resisted sprints. Other researchers using loaded resisted sprints of 35 and 55% v_dec_ indicated no significant differences in both sprint velocity and the kinetics (vertical and horizontal force, vertical and horizontal impulse and rate of force development) of the first step of 5 m sprint measured using a force plate [24]. Mangine et al. [26] also found no improvements in 20 m sprint time and force, velocity and power following resisted sprints using a robotic resistance device (1080 Motion) with 5% BM, but an increase in the rate of force development was observed. In the present study, 9 out of 12 participants showed higher *v*_0_ by an average of 1.2% (ES = 0.43), which indicates trends of higher capabilities of the lower limbs to produce horizontal force at high velocities after performing heavy sled towing. Additionally, a 2.6% improvement in *P_max_* (ES = 0.47) was noticed (7 out of 12 participants improved *P_max_*), which reflects trends of higher capabilities for mechanical power output during the acceleration phase. These observations may partly explain the increase in velocity that occurred following sled towing. A previous study argued that the sled load determines the subsequent adaptions to sprint performance [20]. Heavy sled towing (load > 20% of BM) results in an improvement in the initial acceleration during sprinting, where the force output is high, and velocity is low. Morin et al. [8] examined the effect of 10 weeks of resisted sled training with individual optimal load and found improvements in 5 and 30 m sprint performance and mechanical variables *F*_0_, *v*_0_, *P_max_* and *RF_max_*. This current study showed that sled towing with the individual optimal load leads to a significant decrease in 30 m sprint time along with an increase in velocity during the acceleration phase of the sprint. However, more studies are needed to establish the acute and long-term benefits of optimal load training.

Sprint kinematics of baseline and post-intervention sprints were assessed. Results indicated no significant systematic changes in mean step length and frequency and mean contact and flight time. The improvement in velocity observed after the resisted sprints resulted from an increase in step length and/or step frequency. These kinematic parameters differ among athletes, and therefore, in some participants, the increase in velocity was caused by increasing step length, whereas others showed higher step frequency. In any case, the findings of this study indicated that high load resisted sprints do not impair the running technique of the following unresisted sprint. Limited studies examined the acute effects of resisted sprints on sprint kinematics and present various methodological differences [24,26,27,28,29]. Van Den Tillaar and Von Heimburg [28] investigated the effect of resisted sprints with an absolute load of 5 kg (corresponding to ~7.3% BM) on 20 m sprint performance and kinematic parameters throughout the distance of 20 m. Sprint performance was enhanced by 2%, but step length and frequency and contact and flight time were not significantly changed. Jarvis et al. [27] also showed no significant changes in step length and frequency of the third step in 15 m sprint, although performance was significantly improved. Other studies did not find a significant effect of resisted sprints on both sprint performance and kinematics [24,26,29]. The level of the participants and their technical experience may explain the improvement of the performance but not the corresponding improvement in the kinematic parameters of the sprint. Participants in the studies of Van Den Tillaar and Von Heimburg [28] and Jarvis et al. [27] were players of team sports, while in this study, they were sprinters. However, higher-level sprinters with a stable running technique may have shown different results. Therefore, further research is warranted.

This study has limitations that must be mentioned. Participants could not be controlled for lifestyle variables such as diet, sleep, hydration, school and work that may affect their performance. Furthermore, the F-v profile was determined by the field method proposed by Samozino et al. [3], which has limitations such as estimating the horizontal aerodynamic drag force from only stature, body mass and a fixed drag coefficient and having the assumption of a quasi-null CM vertical acceleration over the acceleration phase of the sprint. Beyond that, the mechanical variables were derived from instantaneous velocity-time-position data observed by high-speed cameras, and the correct determination of the frame corresponding to the start of the sprint is crucial. Additionally, for the calculation of kinematics, the appropriate frame of touchdown and toe-off should be determined. In order to avoid discrepancies in the analysis, all trials were analyzed by the same researcher.

## 5. Conclusions

This study showed that the performance of two resisted sprints of 20 m with a load of 50% v_dec_ of individual *v*_0_ is an effective conditioning stimulus and enhances subsequent 30 m sprint performance. This finding argues that setting an individual load as a percentage of v_dec_ is efficient in eliciting PAP. No significant changes were observed in the mechanical variables; however, the individual analysis revealed some strong treads of improvements in *v*_0_ and *P_max_*. The performance improvement was not accompanied by systematic changes in sprint kinematic characteristics. Future research should include higher-level sprinters to determine if acute alterations in kinematic parameters could be caused following resisted sprints. In conclusion, sled towing with the individual optimal load acutely enhances performance in the acceleration phase of sprint without affecting the athlete’s running technique.

## Figures and Tables

**Table 1:** Mean ± *SD* average values of kinematic variables for 5 m intervals of baseline and post-intervention 30 m sprints for the experimental condition.

Experimental Condition		Velocity (m/s)	Step Length (m)	Step Rate (Hz)	Contact Time (s)	Flight Time (s)
**0–5 m**	Pre	3.55 ± 0.26	1.15 ± 0.08	3.11 ± 0.36	0.175 ± 0.018	0.079 ± 0.010
	Post	3.59 ± 0.24	1.14 ± 0.07	3.15 ± 0.34	0.180 ± 0.023	0.077 ± 0.011
	ES (95% CI)	0.38 (−0.22–0.96)	−0.06 (−0.63–0.50)	0.35 (−0.25–0.92)	0.38 (−0.22–0.96)	−0.30 (−0.87–0.29)
**5–10 m**	Pre	6.69 ± 0.48	1.57 ± 0.08	4.28 ± 0.31	0.143 ± 0.013	0.101 ± 0.009
	Post	6.75 ± 0.53 *	1.57 ± 0.08	4.30 ± 0.34	0.146 ± 0.015	0.102 ± 0.010
	ES (95% CI)	0.71 (0.58–1.33)	0.18 (−0.40–0.75)	0.21 (−0.37–0.77)	0.45 (−0.16–1.03)	0.09 (−0.48–0.66)
**10–15 m**	Pre	7.57 ± 0.61	1.76 ± 0.09	4.31 ± 0.30	0.133 ± 0.012	0.108 ± 0.006
	Post	7.64 ± 0.66 *	1.77 ± 0.09	4.33 ± 0.30	0.133 ± 0.013	0.108 ± 0.007
	ES (95% CI)	0.98 (0.27–1.65)	0.19 (−0.39–0.75)	0.18 (−0.39–0.75)	0.07 (−0.50–0.64)	−0.03 (−0.59–0.54)
**15–20 m**	Pre	7.96 ± 0.72	1.87 ± 0.10	4.27 ± 0.28	0.126 ± 0.012	0.112 ± 0.006
	Post	8.05 ± 0.77 *	1.87 ± 0.10	4.30 ± 0.34	0.126 ± 0.013	0.113 ± 0.006
	ES (95% CI)	1.06 (0.33–1.76)	0.17 (−0.41–0.73)	0.19 (−0.38–0.76)	−0.17 (−0.74–0.40)	0.18 (−0.40–0.74)
**20–25 m**	Pre	8.19 ± 0.79	1.93 ± 0.11	4.25 ± 0.29	0.121 ± 0.012	0.117 ± 0.006
	Post	8.29 ± 0.85 *	1.94 ± 0.12	4.27 ± 0.25	0.121 ± 0.013	0.117 ± 0.005
	ES (95% CI)	0.83 (0.16–1.48)	0.30 (−0.29–0.81)	0.24 (−0.34–0.81)	0.03 (−0.54–0.60)	−0.02 (−0.55–0.58)
**25–30 m**	Pre	8.37 ± 0.87	1.98 ± 0.12	4.20 ± 0.30	0.120 ± 0.011	0.122 ± 0.007
	Post	8.46 ± 0.92 *	2.00 ± 0.11	4.21 ± 0.29	0.120 ± 0.014	0.122 ± 0.006
	ES (95% CI)	0.84 (0.16–1.49)	0.43 (−0.18–1.01)	0.11 (−0.46–0.67)	−0.15 (−0.72–0.42)	−0.11 (−0.67–0.46)

* *p* < 0.05 significantly different from baseline trial.

**Table 2:** Mean ± *SD* average values of kinematic variables for 5 m intervals of baseline and post-intervention 30 m sprints for the control condition.

Control Condition		Velocity (s)	Step Length (m)	Step Rate (Hz)	Contact Time (s)	Flight Time (s)
**0–5 m**	Pre	3.55 ± 0.20	1.16 ± 0.08	3.08 ± 0.30	0.175 ± 0.022	0.082 ± 0.010
	Post	3.56 ± 0.24	1.15 ± 0.09	3.12 ± 0.35	0.175 ± 0.019	0.081 ± 0.011
	ES (95% CI)	0.09 (−0.48–0.65)	0.17 (−0.73–0.41)	0.26 (−0.32–0.83)	−0.10 (−0.47–0.67)	−0.20 (−0.77–0.38)
**5–10 m**	Pre	6.75 ± 0.50	1.58 ± 0.09	4.27 ± 0.32	0.144 ± 0.013	0.101 ± 0.011
	Post	6.71 ± 0.49	1.58 ± 0.09	4.25 ± 0.31	0.144 ± 0.013	0.103 ± 0.011
	ES (95% CI)	−0.31 (−0.88–0.28)	0.02 (−0.55–0.58)	−0.23 (−0.80–0.35)	0.09 (−0.48–0.65)	0.30 (−0.29–0.87)
**10–15 m**	Pre	7.65 ± 0.64	1.77 ± 0.10	4.32 ± 0.27	0.132 ± 0.011	0.107 ± 0.007
	Post	7.61 ± 0.61	1.77 ± 0.09	4.29 ± 0.29	0.131 ± 0.012	0.110 ± 0.007
	ES (95% CI)	−0.28 (−0.86–0.30)	0.11 (−0.47–0.67)	−0.55 (−1.15–0.69)	−0.13 (−0.70–0.44)	0.59 (−0.04–1.11)
**15–20 m**	Pre	8.04 ± 0.73	1.88 ± 0.11	4.27 ± 0.27	0.125 ± 0.011	0.113 ± 0.005
	Post	8.01 ± 0.73	1.88 ± 0.12	4.25 ± 0.28	0.126 ± 0.012	0.114 ± 0.007
	ES (95% CI)	−0.22 (−0.79–0.28)	0.08 (−0.49–0.65)	−0.19 (−0.75–0.39)	0.10 (−0.47–0.66)	0.25 (−0.33–0.82)
**20–25 m**	Pre	8.26 ± 0.79	1.95 ± 0.11	4.24 ± 0.27	0.123 ± 0.011	0.117 ± 0.005
	Post	8.23 ± 0.79	1.94 ± 0.11	4.24 ± 0.27	0.121 ± 0.012	0.118 ± 0.006
	ES (95% CI)	−0.34 (−0.91–0.25)	−0.20 (−0.77–0.37)	0.02 (−0.59–0.55)	−0.46 (−1.05–0.14)	0.03 (−0.54–0.59)
**25–30 m**	Pre	8.42 ± 0.83	2.00 ± 0.10	4.20 ± 0.26	0.119 ± 0.014	0.122 ± 0.006
	Post	8.39 ± 0.84	2.01 ± 0.13	4.17 ± 0.29	0.120 ± 0.011	0.122 ± 0.007
	ES (95% CI)	−0.32 (−0.89–0.27)	0.05 (−0.52–0.62)	−0.39 (−0.97–0.21)	0.23 (−0.35–0.80)	0.24 (−0.34–0.81)

**Table 3:** Mean ± *SD* values of mechanical variables of baseline and post-intervention 30 m sprints for the two conditions.

Mechanical Variables	Experimental Condition			Control Condition		
	Pre	Post	ES (95%CI)	Pre	Post	ES (95%CI)
*F*_0_ (N·kg^−1^)	7.51 ± 0.63	7.60 ± 0.66	0.16 (−0.41–0.73)	7.72 ± 0.84	7.61 ± 0.60	−0.19 (−0.76–0.38)
*v*_0_ (m·s^−1^)	9.02 ± 1.11	9.13 ± 1.15	0.43 (−0.17–1.01)	9.07 ± 1.03	9.03 ± 1.05	−0.30 (−0.87–0.29)
*P_max_* (W·kg^−1^)	17.02 ± 3.01	17.47 ± 3.46	0.47 (−0.14–1.06)	17.62 ± 3.51	17.30 ± 3.15	−0.25 (−0.82–0.33)
*S_Fv_* (N·s^−1^/m·kg)	−0.84 ± 0.09	−0.84 ± 0.07	0.29 (−0.54–0.59)	−0.86 ± 0.09	−0.85 ± 0.06	0.13 (−0.44–0.70)
*RF_max_* (%)	44.4 ± 3.0	44.9 ± 3.6	0.43 (−0.17–1.01)	45.1 ± 3.6	44.8 ± 3.2	−0.23 (−0.80–0.35)
*D_RF_* (%·s·m)	−7.8 ± 0.9	−7.8 ± 0.7	0.09 (−0.48–0.65)	−7.9 ± 0.9	−7.8 ± 0.7	0.15 (−0.42–0.72)

*F*_0_ = theoretical maximal horizontal force, *v*_0_ = theoretical maximal horizontal velocity, *P_max_* = theoretical maximal horizontal power, *S_Fv_* = slope of the linear F–v relationship, *RF_max_* = maximal value of ratio of horizontal force to total ground reaction force, *D_RF_* = decrease in *RF* with increasing velocity.

## Data Availability

The data presented in this study are available from the corresponding author on reasonable request.
